# Deep Learning-based Non-rigid Image Registration for High-dose Rate Brachytherapy in Inter-fraction Cervical Cancer

**DOI:** 10.1007/s10278-022-00732-6

**Published:** 2022-11-23

**Authors:** Mohammad Salehi, Alireza Vafaei Sadr, Seied Rabi Mahdavi, Hossein Arabi, Isaac Shiri, Reza Reiazi

**Affiliations:** 1 Department of Medical Physics, School of Medicine, Iran University of Medical Sciences, Tehran, Iran; 2 Department of Theoretical Physics and Center for Astroparticle Physics, University of Geneva, Geneva, Switzerland; 3 Institute of Pathology, RWTH Aachen University Hospital, Aachen, Germany; 4 Division of Nuclear Medicine and Molecular Imaging, Geneva University Hospital, CH-1211 Geneva 4, Switzerland; 5 Division of Radiation Oncology, Department of Radiation Physics, University of Texas MD Anderson Cancer Center, Houston, USA

**Keywords:** Locally advanced cervix cancer, Deformable image registration, Brachytherapy, Convolutional neural networks, CT

## Abstract

In this study, an inter-fraction organ deformation simulation framework for the locally advanced cervical cancer (LACC), which considers the anatomical flexibility, rigidity, and motion within an image deformation, was proposed. Data included 57 CT scans (7202 2D slices) of patients with LACC randomly divided into the train (*n* = 42) and test (*n* = 15) datasets. In addition to CT images and the corresponding RT structure (bladder, cervix, and rectum), the bone was segmented, and the coaches were eliminated. The correlated stochastic field was simulated using the same size as the target image (used for deformation) to produce the general random deformation. The deformation field was optimized to have a maximum amplitude in the rectum region, a moderate amplitude in the bladder region, and an amplitude as minimum as possible within bony structures. The DIRNet is a convolutional neural network that consists of convolutional regressors, spatial transformation, as well as resampling blocks. It was implemented by different parameters. Mean Dice indices of 0.89 ± 0.02, 0.96 ± 0.01, and 0.93 ± 0.02 were obtained for the cervix, bladder, and rectum (defined as at-risk organs), respectively. Furthermore, a mean average symmetric surface distance of 1.61 ± 0.46 mm for the cervix, 1.17 ± 0.15 mm for the bladder, and 1.06 ± 0.42 mm for the rectum were achieved. In addition, a mean Jaccard of 0.86 ± 0.04 for the cervix, 0.93 ± 0.01 for the bladder, and 0.88 ± 0.04 for the rectum were observed on the test dataset (15 subjects). Deep learning-based non-rigid image registration is, therefore, proposed for the high-dose-rate brachytherapy in inter-fraction cervical cancer since it outperformed conventional algorithms.

## Introduction

Cervical cancer is one the most common cause of cancer-related female mortality in the world [1] and locally advanced cervical cancer (LACC) is the most common type of cervix cancer [2] which is normally external beam radiation therapy (EBRT) and brachytherapy (BT) and chemotherapy were used to treat the patient. The combination of EBRT and chemotherapy is usually the first step in delivering a prescribed dose to the planning target volume. BT is the second component of cervical cancer treatment, and it involves using low- or high-dose radioactive sources to irradiate the residual tumor locally. The applicators usually set the sources near to the target in order to deliver the maximal dose while protecting normal/healthy tissues. The large inter-fraction organ deformations occur during the BT treatment (different treatment sessions) which causes noticeable uncertainties in the estimated cumulative dose over the entire treatment course [3, 4]. For instance, target deformations of 20 mm and 48 mm have been reported for the cervix [5] and uterus [6], respectively. Moreover, some studies have reported an average of 63% tumor shrinkage during the treatment [7–10]. Dosimetry uncertainties for organs at risk (OARs) have been reported to be 20–25% in intra- and inter-fraction D2cc, indicating that organ deformation contributes significantly to ORAs dosage errors [11–13]. For toxicity prediction, some research advocated using a deformable image registration (DIR) approach to map the EBRT dose to the spatial coordinate of the initial BT percentage [14–16]. Intensity-based image alignment methods (e.g., Demons algorithms) are not an optimal solution for DIR for cervical cancer BT due to large and low deformations in the different organs and tissue contrast, respectively [13, 17].

Recently, deep learning approaches, including supervised, unsupervised, and semi-supervised methods, are employed in different medical image analysis tasks [18–28], different BT tasks [29–31], and learn and carry out spatial alignment/transformation between images [32–41]. These methods usually used convolutional neural networks (CNNs) to extract informative features automatically to perform this task [32–41]. In these studies, different versions of CNN models are trained to learn small sets of transformation parameters, such as translation, rotation, scaling, and affine transformations, in order to offer the optimal transformation parameters for a perfect alignment of the two images. In other words, the output of the models would be a transformation matrix rather than transformed images. On the other hand, CNN models can be trained to directly perform image deformation/alignment on the input image. In a study conducted by Miao et al. [37], a CNN model was used to estimate transformation parameters in a one-shot for rigid registration for 2D cone-beam CT to CT images. Similarly, Cao et al. [32] trained a CNN model to predict transformation parameters in a thin-palate spline for the DIR of brain MRI images. Eppenhof et al. [33] employed a 3D CNN model to learn thin-plate spline transformation parameters between pairs of 3D images. Sokooti et al. [41] proposed a CNN model which could directly predict a dense displacement vector field (DVF) from a pair of input images. Similar to other supervised deep learning tasks, the quality and quantity of the training dataset play a key role in the overall performance of the model.

Alternatively, unsupervised deep learning algorithm-based solutions are more appealing for this type of challenge. For example, Wu et al. [38] proposed a convolutional stacked auto-encoder (CAE) framework to extract discriminative features from pairs of fixed and moving images. They employed the Vercauteren et al. [42] and Shen et al. [43] methods to improve the registration on brain MR images. Though the CAE is an unsupervised method, it has been optimized for image reconstruction applications rather than dedicatedly for image registration, as such there is no guarantee that the extracted features are optimal for image registration. Dosovitskiy et al. [44] employed an unsupervised CNN model to estimate the optical flow across the video frames. This study addressed a 2D video sequencing, which contained relatively low levels of noise (compared to medical images), high contrast, and a relatively small deformation between adjacent frames. Medial pictures, on the other hand, have a lot of noise, little tissue contrast across organs of interest, and may require a lot of 3D image modification. As mentioned earlier, in fully supervised transformation estimation, the creation of ground-truth data to train the deep learning models is the most challenging task in the deformable medical image registration problem.

However, for the DIR problem, a dense flow field ground-truth correspondence is rarely available. Some studies proposed specific frameworks to generate synthesized deformation parameters for the creation of a training dataset for the tasks of rigid registration [37], non-rigid image registration [33, 41], and manual annotation [43]. Unlike these methods, Uzunova et al. [45] used statistical appearance models to generate a ground-truth dataset in a 2D fashion for supervised training in the brain and cardiac imaging. Real time image registration could be achieved by using this approach; however, this method has several challenges, such as preparing the ground-truth or the training dataset with clinical considerations.

In this study, three major issues are addressed: (1) simulation of a realistic local deformation in the image domain that takes into account clinical considerations. As mentioned above, defining a realistic deformation, especially locally and/or organ-wise, would be a challenging task for the creation of the registration ground-truth dataset. In this regard, a novel training data generation framework is proposed in this study, which enables the definition of any desirable deformations depending on the organ rigidity/mobility. (2) Creation of a dataset for DIR problems, which can be used by any machine learning approach. (3) Development of a multi-channel deep neural network to simultaneously process the original CT images together with the binary masks of the OARs.

This study proposes a new method for the inter-fraction deformation simulation in the HDR brachytherapy to be used in a deep learning-based DIR algorithm. Furthermore, a deep learning-based DIR algorithm is developed to directly perform the image alignment of the inter-fraction HDR sessions. A deep learning model consisting of five input channels was implemented and dedicated to the CT images of the bladder, cervix, rectum, and bone OARs in the form of binary masks to perform image alignment between two CT slices.

## Methods

### Dataset

Since there is no publicly available training dataset specific for the task of image alignment, a large dataset of the female pelvis was collected for the inter-fraction deformation simulation. Fifty-seven patients with cervical cancer diagnosed at the cancer institute of this study between 2017 and 2018 were included in this study. The collected data included planning CT images and the corresponding RT structure data. For each patient, a CT scan was acquired in the supine position from about L5 vertebrae to the femoral heads. All images were taken by the same CT scanner (SOMATOM Scope CT scan, Siemens Healthcare, Forchheim, Germany). All CT images were acquired with the same acquisition protocol (KVp = 110 and mAs = 130). Image dimension was set at 512 × 512 for each trans-axial slice with about 80–150 slices with a pixel spacing of 0.84 mm and a slice thickness of 5 mm. In total, there were 7202 2D CT images. The RT structure data, in the form of manually defined contours for each organ, were delineated by a physician for the bladder, rectum, and cervix. The study protocol was approved by the ethical guidelines. The datasets of 57 patients were randomly divided into a 42-patient training set and a 15-patient test set.

### Image Pre-processing

#### Binary Mask from RT Structures

**Fig. 1:**
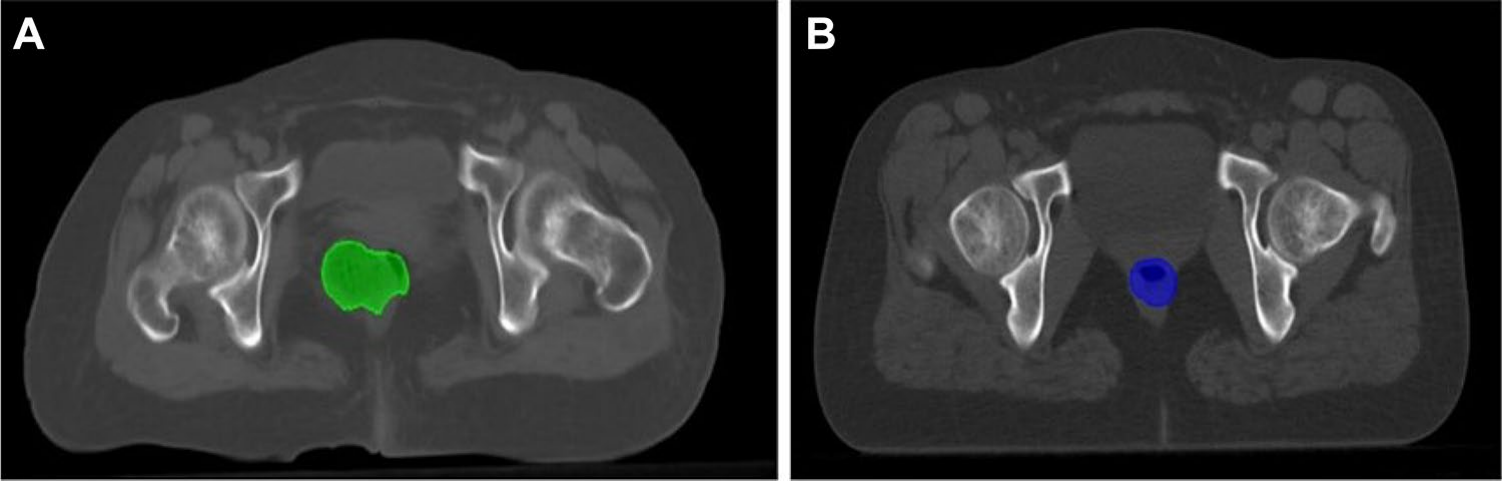
Representative trans-axial CT slices of two different patients showing the different rectum volume/location between two sets of CT images

Due to the inter-patient anatomical structure variations and intra-patient anatomy motion, manual organ delineations were employed from the RT structure data to guide local image deformation. A representative sample of rectum deformation between two sets of CT images is presented in Fig. 1.

##### Bone Extraction

In the first step, the binary masks of the key organs in the pelvis region, including the rectum, bladder, and cervix, were obtained. It was also necessary to consider a binary mask of the bony structures for the simulation of image deformation. The binary masks of the bony structures were obtained from CT images by an intensity-based segmentation on the CT number (HU > 150). The segmented bony structures were visually verified and manually corrected to avoid any mis-segmentation errors.

##### Couch Elimination

**Fig. 2:**
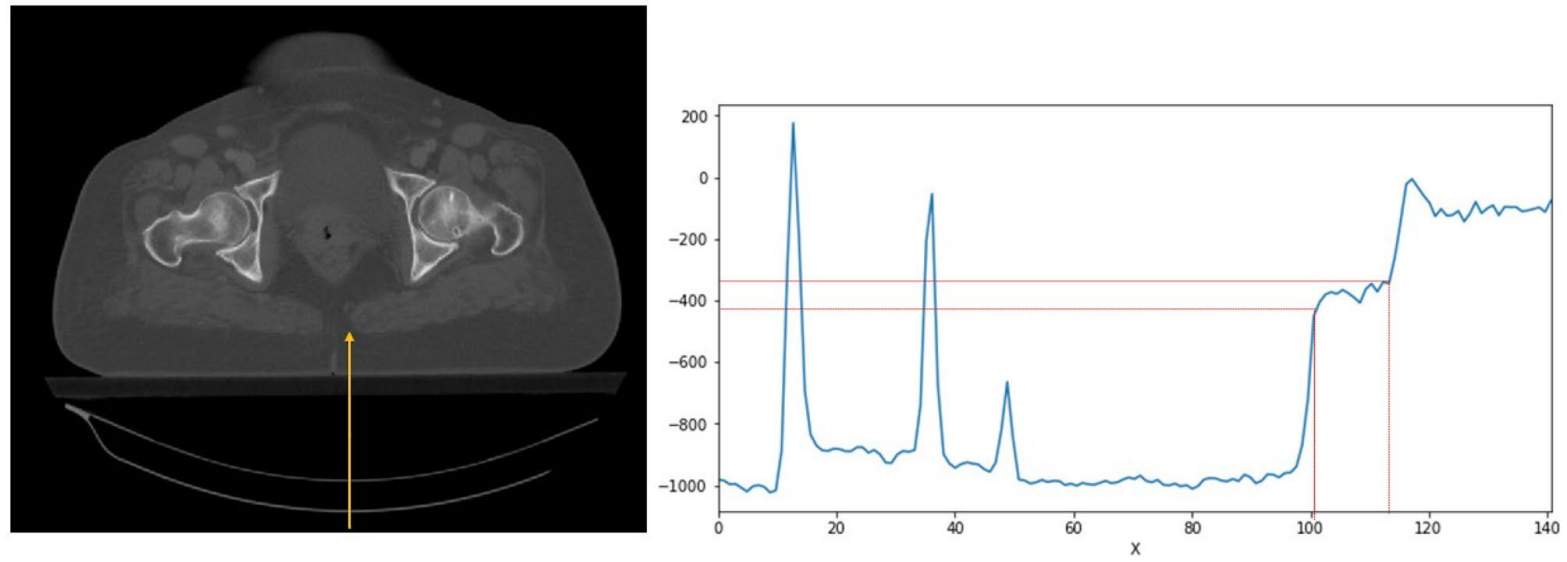
Intensity-based thresholding approach was employed to remove couches from CT images. Left: an example of a 2D CT image with a line profile in the posterior-anterior direction. Right: CT intensity profile along the line depicted on the CT image shows the specific intensity of the CT couch

One of the main steps in data preparation for the deep learning models is to remove non-related components/regions from the input images to enhance the overall efficiency of the model. To this end, the couches were removed from the input CT images by applying an intensity-based thresholding method. To remove the couches from the entire slices, a CT intensity profile in the posterior-anterior direction was drawn to detect the coach intensity ranges (− 405 HU < couch intensity <  − 340 HU) (Fig. 2). The couch removal was visually verified to avoid any mis-segmentation errors.

Furthermore, the original CT images, stored in a 512 × 512 matrix, were cropped to a 256 × 512 matrix to fit the body contour in order to enhance memory and computational efficiencies and image were checked to avoid removing body regions in cropped images.

#### Image Deformation Simulation

##### Deformation Considerations

**Fig. 3:**
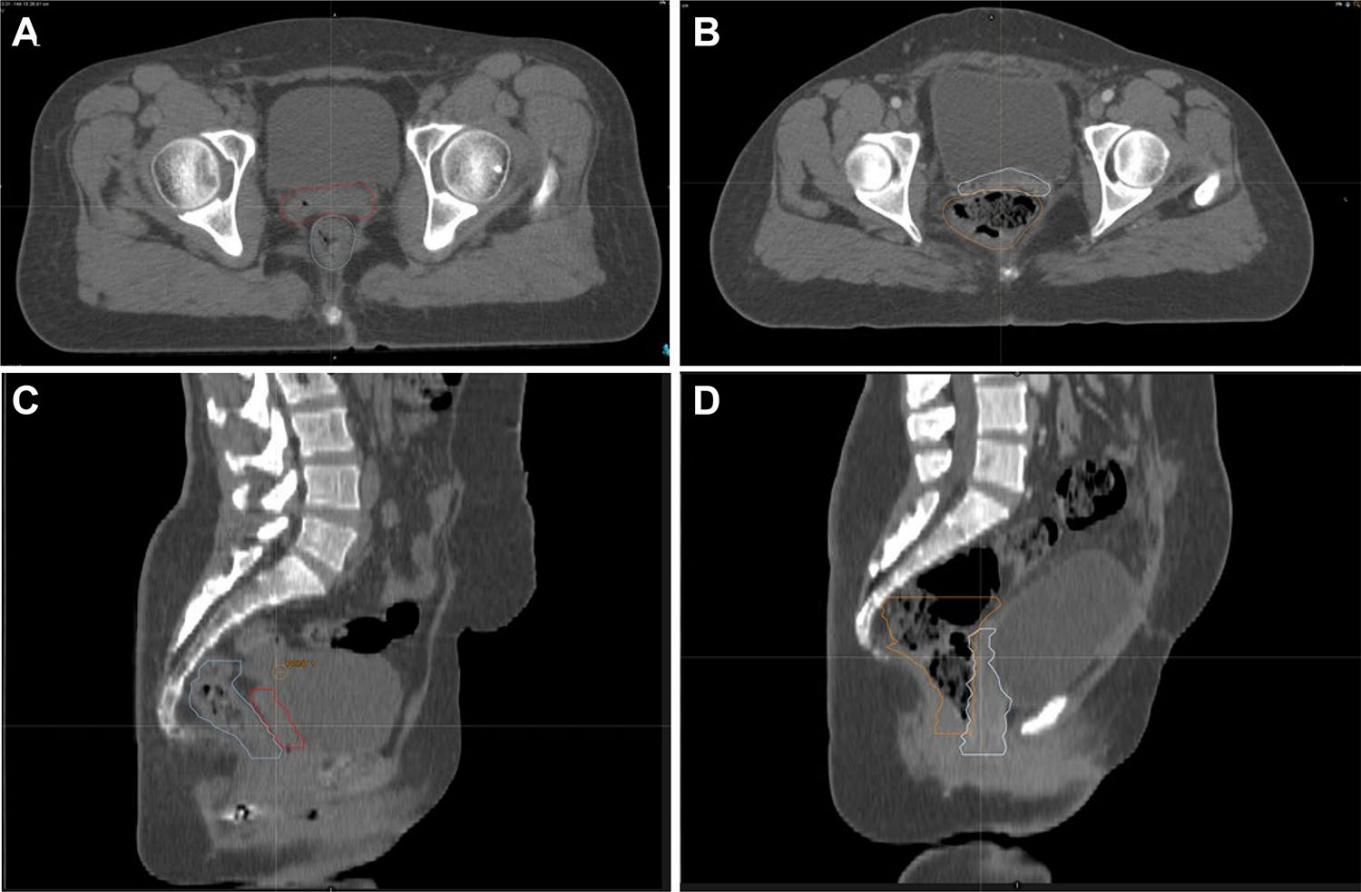
A representative example of cross-organ deformation is shown wherein a rectum filling has greatly impacted the cervix. The trans-axial and sagittal CT views of two patients with different rectum filling are shown. In **A** and **C**, the rectum with a normal filling has not affected the cervix. In **B** and **D**, the rectum filling has substantially deformed the cervix

Generally, bony structures do not have any significant deformation when the applicator is inserted into the vaginal cavity. On the other hand, flexible/soft tissue organs may undergo noticeable deformation after the insertion of the applicator, usually very locally around the cervix region [3]. The cervix is a flexible organ which might be non-rigidly deformed depending on the patient’s posture. The bladder and rectum are also highly flexible organs which could be filled or empty within the course of therapy and may also cause deformation to the surrounding organs. The bladder and rectum are regarded as OARs while the cervix is the target organ for dose delivery [46]. For a realistic simulation of the organ deformation in the pelvis regions, certain criteria should be met [3–5, 47, 48]. The uterus is a flexible organ which is prone to both rotational and translational motions [5]. Moreover, the uterine and vaginal deformation/motion also depend on the status of the bladder and rectum (whether they are empty or not) [5]. The cross-organ deformation impacts vary greatly across different organs, as illustrated in Fig. 3 wherein the rectum filling has remarkably deformed the cervix, compared to the bladder filling which had less impact.

##### Deformation Simulation

To implement image deformation, the correlated stochastic field [49] was simulated using the same size as the target image (used for deformation) to produce general random deformation. Input images were patients’ CT scans and the target images were deformed images using the abovementioned method. For a Gaussian stochastic deformation (*Y*_*G*_) with an average of *y*_*c*_ and a dispersion of $\sigma$, the probability for *Y*_*G*_ = [*y*, *y* + *dy*] is given by Eq. (1):

1 $$p\left(y\right)dy=\frac{1 }{\sqrt{2\pi {\sigma }^{2}}}\mathrm{exp}\left\{-\frac{{\left(y-{y}_{c}\right)}^{2}}{2{\sigma }^{2}}\right\}dy$$

In such a Gaussian random field, the probability for the field at location × 1 would have a value of *Y*(× 1) = *y*1 at location × 2 and a value of *Y*(× 2) = *y*2, and so on for *N* points (i.e., for *Y*(*xN*) = *yN* at location *xN*). For a Gaussian field knowledge of all *n* points, the probability distributions would be:

2 $$p ({y}_{1, }\dots .,{y}_{n)}d{y}_{1}\dots d{y}_{n}=\frac{1}{2\pi {\left(\mathrm{det}M\right)}^{1/2}}\mathrm{exp}\{-\frac{\left({y}_{1, }\dots .,{y}_{n}\right){M}^{-1 }\left(\begin{array}{c}{y}_{1, }\\ \dots \\ {y}_{n, }\end{array}\right)}{2}\}d{y}_{1}\dots d{y}_{n}$$

In which *M*^−1^ is the inverse of the correlation matrix *M*:

$$M=\left(\begin{array}{cc}\xi (0)& \xi ({y}_{1},{y}_{2})\\ \xi ({y}_{1},{y}_{2})& \xi (0)\end{array}\right)=\left(\begin{array}{cc}{\sigma }^{2}& \xi (0)\\ \xi (0)& {\sigma }^{2}\end{array}\right)$$

In which $\xi$ is the covariance function.

**Fig. 4:**
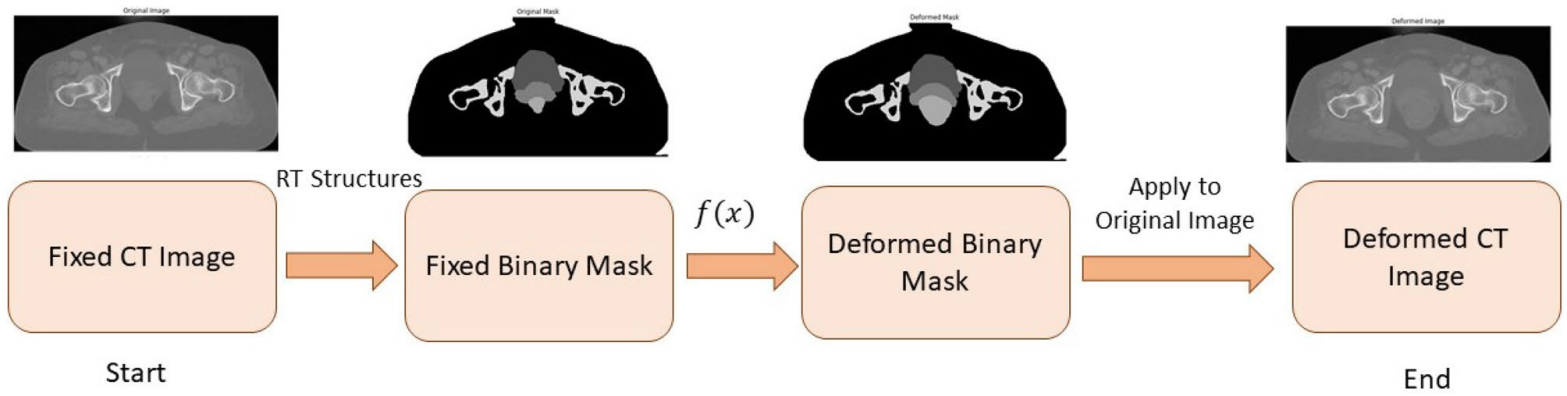
Synthetic image deformation workflow. From left to right: input CT image was considered a fixed image, the binary masks of the key organs (created from the RT structure data) were defined on the fixed image, a non-linear deformation was applied on the binary mask, and subsequently, a deformed CT image was created from the deformed binary masks

The simulated deformation field moves all regions with the same strength. To add anatomical considerations, the field strength in bony structures and air (outer body) regions was decreased using regional information and a smoothed correction map. Afterward, the rectum expansion simulation was added, which is a random expansion modulated on the general deformation field. For the deformation offset field, a sigma ($\sigma$) of 10 was chosen while for the bony structure, $\sigma =3$ was used. Furthermore, to take into account the anatomical consideration in deformation offsets for the rectum field, the deformation amplitude was set within the range of 100 to 1000 and for the bladder, 100 to 500 were chosen. Next, a non-linear wrap was applied to the input image, in which the wrap field was specified by offset vectors which define the correspondence between pixel values in the source and the output images at the same original location. The pixel value was obtained by the bilinear interpolation of the 4 neighboring pixels. Additionally, for pixels residing outside the considered ROI (i.e., the ROIs are binary masks specified for the OARs), the nearest pixel values at the corresponding mask boundary were calculated using an image wrapping based on the per-pixel flow vectors. A dense image wrapping takes a 4D shape tensor (batch, height, width, and channels), as well as an offset 4D tensor (batch, height, width, channel) as inputs, and returns a 4D float tensor (batch, height, width, and channels). Therefore, applying this deformation on either images or masks would allow the local deformation of an image considering anatomical condition/consideration (Fig. 4).

**Fig. 5:**
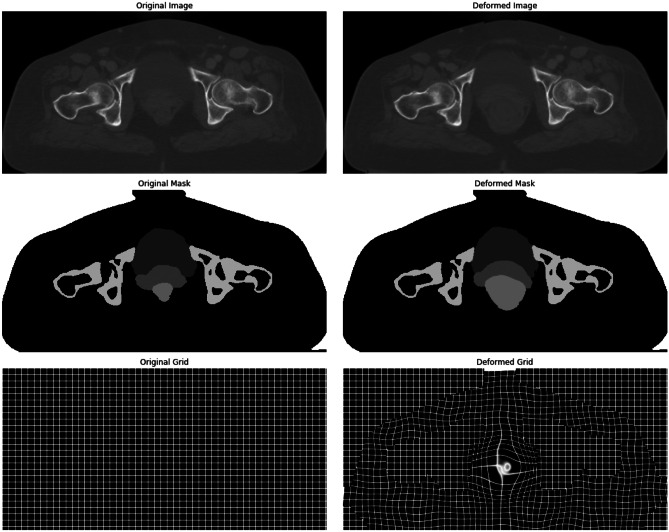
First row: original and deformed images. Second row: original and deformed masks. Third row: original and deformed grids/fields

As shown in Fig. 5, bony structures did not change in the output deformed image after applying the image deformation. This deformation field was optimized to have a maximum amplitude in the rectum region, a moderate amplitude in the bladder region, and an amplitude as minimum as possible within bony structures.

### Deep Neural Network Architecture

The DIRNet deep learning model proposed by de Vos et al. [50] was used as a baseline model. It is an end-to-end CNN that consists of convolutional regressors, spatial transformation, and resampling block. The convolutional regressor receives concatenated pairs of fixed images (as input) and employs 4 alternating convolutional layers with a kernel size of 3 × 3 and 0-padding followed by 2 × 2 down sampling layers.

**Fig. 6:**
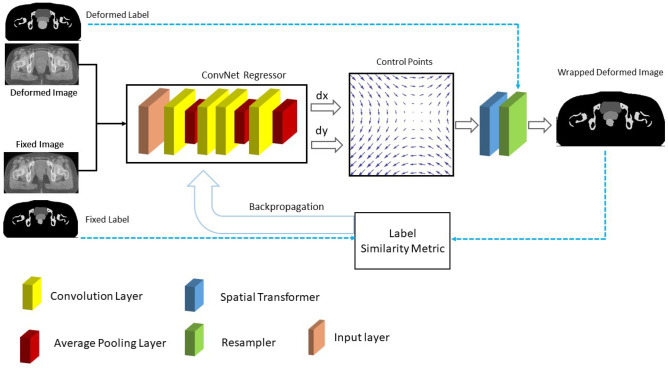
Training workflow of the DIRNet architecture. The DIRNet model takes a concatenated pair of fixed and deformed images as inputs. The convolutional regressor generates a grid of control points by analyzing the spatial correspondence between the input images. The spatial transformer generates a displacement vector field that enables the ReSampler to wrap the moving image to the fixed image. Solid lines indicate the baseline DIRNet and the blue dash-line indicates the proposed modification for the loss calculation in the backpropagating flow

The spatial transformer creates a displacement vector field (DVF) that allows the resampler to wrap a moving image around a fixed image. The wrapped image is compared to the fixed image through a normalized cross-correlation (NCC) as a similarity metric which was employed as the loss function. The DIRNet is trained through optimizing a backpropagating dissimilarity between pairs of moving and fixed images using the mini-batch stochastic gradient descent (Adam) algorithm [51] (Fig. 6).

#### ConvNet Regressor

The baseline DIRNet model utilized a Convolutional Net Regressor which consists of 4 layers of $3\times 3$ convolutional (Conv) layer with a 0-padding and average pooling layers with a size of $2\times 2$ to retain the underlying information during the down-sampling. Sixteen kernels were used per Conv layer. All Conv layers used exponential linear units (ELU) [52] as an activation function, except for the last layer, which had a linear output. ConvNet Regressor expected a concatenated pair of fixed and deformed images as input. In addition, a batch normalization [52] with a momentum of 0.9 was applied for each layer.

#### Spatial Transformer

The spatial transformer introduced by Jaderberg et al. [34], a differentiable attention to any spatial transformation, can be trained using a backpropagation. The spatial transformer generates a dense DVF for local deformation parameters produced by ConvNet Regressor and allows a neural network to learn how to perform a spatial transformation on the input image to enhance the geometric invariance of the model. In this model, a cubic B-spline transformer was employed to its local support capability as a spatial transformer. It takes into account the data points in the neighborhood to modify the location of the target point leading to a continuous/smooth and natural deformation effect. According to Eq. (3), in the case of 2D, *f*(*x*) would depend on a couple of control points ($x$ to update the location of the target point ($\varnothing (x-i)$)).

3 $$f\left(x\right)={\sum }_{i=\mathrm{1,2},3}{\alpha }_{i}\varnothing (x-i)$$

#### ReSampler

The DVF generated by the spatial transformer needs to be applied to the moving image to transform it into the space of the fixed image. This process requires resampling the moving image on the grid belonging to the pixels (voxels) in the fixed image. Since a local deformation was used, the bicubic interpolation was employed to transform the moving images.

### Network Training and Implementation Details

Different types of DIRNet were trained to investigate the impact of various DIRNet parameters, such as the loss function, as well as the number of Conv layers and the max-pooling layers. First, to evaluate the effect of the loss function, the DIRNet 2 was designed with the mean square error (MSE) loss function. Second, to show the effect of the down-sampling layer, the DIRNet 3 was designed without an average pooling. Third, to evaluate the effect of the number of Conv layers, the DIRNet 4 with 8 Conv layers were designed and implemented. Table 1 summarizes the baseline DIRNet model and its variant properties.

**Table 1:** Key parameters of the baseline DIRNet and all its variants

Models	Number of layers	Number of trainable parameters	Kernel size	Loss function	Pooling layer	Activation function	Batch normalization
DIRNet 1 (baseline)	4	446,722	3 × 3	Normalized cross-correlation	Yes (2 × 2)	ELU	✔
DIRNet 2	4	446,722	3 × 3	Mean square error	Yes (2 × 2)	ELU	✔
DIRNet 3	4	815,874	3 × 3	Normalized cross-correlation	No	ELU	✔
DIRNet 4	8	446,722	3 × 3	Normalized cross-correlation	Yes (2 × 2)	ELU	✔

ELU Exponential Linear Units

Furthermore, for the training of the DIRNet and its variants, the binary masks in the loss function were incorporated for the calculation of backpropagation flows. For this purpose, all DIRNet models take a pair of concatenated fixed and moving images as input. The extracted DVF was also applied to the moving binary mask to produce a wrapped binary mask. The loss function tries to minimize the image dissimilarity error between the wrapped and fixed binary masks to maximize image similarity between fixed and deformed images (Fig. 6).

In the present experiments, the input image was of 256 × 512 voxel size. The 2D Convs were applied followed by the ELU activation functions, except for the final layer which had a linear output with a kernel size of 3 × 3. The down sampling layer had an average pooling layer of 2 × 2 and the batch normalization was applied to every layer. A mini batch of size 4 was utilized since the image size was large. Each model was trained using 10,000 iterations through optimizing (employing mini-batch stochastic gradient descent) image similarity metrics (MSE for the DIRNet 2 and the normalized cross-correlation for the others) as the loss function (comparing the model outputs and fixed images). The learning rate was set at 1e − 4 for all models.

Different packages and libraries were employed, such as the TensorFlow (version 1.13.2) for implementing deep learning models, the Matplotlib^1^ (version 3.1.2) for plotting and visualization, the Scikit-Image^2^ (version 0.15.0) for image processing, and the Numpy^3^ package (version 1.16.4) for numerical computing. All models were trained on a 2 NVIDIA® GTX 1080 GPU and Intel Core i9 Xeon CPU with 128 GB RAM.

### Performance Measurement

After training, the accuracy of each DIRNet model was evaluated by three different similarity metrics. The trained models were applied to the concatenated pair of fixed and moving images to extract the DVF. Next, the extracted DVF was applied to the moving binary mask to wrap it into the fixed binary mask. As such, each organ could be evaluated separately with pixel-level precision. Each similarity measurement score was averaged over the entire patient slices. For the entire OARs (bladder, cervix, and rectum), three different similarity metrics, including two boundary-based and one surface-based metric, were calculated for each slice separately. Afterward, its average was calculated over the entire 2D slices. The mean ± SD calculated over the 2D slices are reported for each of the abovementioned metrics.

#### Jaccard Coefficient

The intersection-over-union, also known as the Jaccard index Eq. (4), was employed as follows:

4 $$Jaccard=\frac{|A\cap B|}{|A\cup B|}$$

where *A* and *B* are the binary masks of the OARs in the fixed and deformed images, respectively.

#### Dice Coefficient Similarity Metric (DC)

The Dice coefficient is very similar to the Jaccard index Eq. (5).

5 $$DC=\frac{2\left|A\cap B\right|}{\left|A\right|+\left|B\right|}$$

#### Average Symmetric Surface Distance (ASSD)

Average symmetric surface distance (ASSD) is a surface distance-based measure. According to Eq. (6), the ASSD measures the average of all the distances from the boundary between two surfaces of the reference and predicted segmentations:

6 $$ASSD=\frac{1}{\left|{B}_{p}+\left|{B}_{G}\right|\right|}\times \left(\sum_{x\epsilon {B}_{P}}d(x,{B}_{G})+\sum_{y\in {B}_{G}}d(y,{B}_{p})\right)$$

where *B*_*p*_ and *B*_*G*_ represent a set of voxels belonging to boundary contours of the predicted mask and ground-truth, respectively. $d(x,{B}_{G})$ and $d(y,{B}_{p})$ are the Euclidean distances between distinct voxels (*y* and *x*) from the ground-truth and predicted contours, respectively [53].

## Results

Tables 1, 2, and 3 present the mean ± SD of the Dice, Jaccard, and the average symmetric distance for the different DIRNet models, respectively, calculated across the 15 patients in the external validation dataset. For the Dice and Jaccard, a higher value indicates a better deformation accuracy. For average symmetric distance, lower values indicate better agreement between the contours of the two corresponding images. Registration performance of the basic DIRNet model and its variants were compared to the conventional iterative intensity-based image registration implemented in the Elastix [54]. To compare the conventional registration algorithm with the DIRNet, a similar grid spacing setting and NCC were chosen. Furthermore, stochastic gradient descent was used for the iterative optimization. Registration was performed in 500 iterations. Moreover, the Gaussian smoothing image Pyramid and multiresolution approach were chosen.

**Table 2:** Dice coefficients calculated over deformed pelvic masks and the ground-truth masks for the different variants of DIRNet model. Results are given for all DIRNet models for the cervix, bladder, and rectum

		Dice coefficient		
		Cervix (mean ± SD)	Bladder (mean ± SD)	Rectum (mean ± SD)
Before registration		0.75 ± 0.14	0.91 ± 0.05	0.65 ± 0.16
SimpleElastix		0.80 ± 0.06	0.90 ± 0.03	0.78 ± 0.09
DIRNet	Base model	0.86 ± 0.08	0.95 ± 0.01	0.87 ± 0.20
	2	0.83 ± 0.08	0.89 ± 0.02	0.74 ± 0.22
	3	0.79 ± 0.06	0.91 ± 0.02	0.79 ± 0.85
	4	**0.89 ± 0.02**	**0.96 ± 0.01**	**0.93 ± 0.02**

**Table 3:** Jaccard coefficients calculated over deformed pelvic masks and the ground-truth masks for different variants of DIRNet models. Results are given for all DIRNet models for the cervix, bladder, and rectum

		Jaccard coefficient		
		Cervix (mean ± SD)	Bladder (mean ± SD)	Rectum (mean ± SD)
Before registration		0.62 ± 0.15	0.85 ± 0.06	0.51 ± 0.19
SimpleElastix		0.71 ± 0.08	0.83 ± 0.04	0.67 ± 0.11
DIRNet	Base model	0.82 ± 0.06	0.91 ± 0.03	0.78 ± 0.07
	2	0.76 ± 0.10	0.81 ± 0.04	0.63 ± 0.23
	3	0.72 ± 0.08	0.85 ± 0.04	0.66 ± 0.10
	4	**0.86 ± 0.04**	**0.93 ± 0.01**	**0.88 ± 0.04**

### Performance on Cervix

**Fig. 7:**
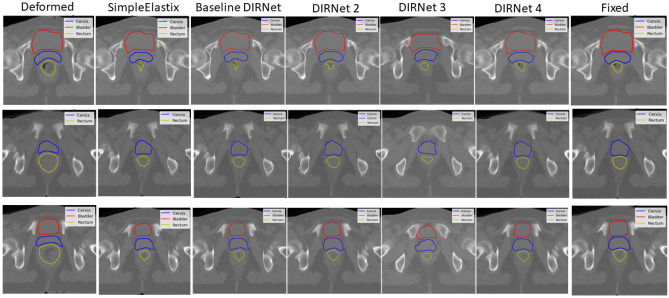
Representative slices of the registered image by the different DIRNet models, in comparison with the conventional intensity-based image registration (SimpleElastix). Red, blue, and yellow contours correspond to the bladder, cervix, and rectum, respectively

The DIRNet 4 model was able to achieve a higher Dice, Jaccard, and lower average symmetric surface distance among all other DIRNet variants evaluated across 15 patients. On average, the DIRNet 4 model achieved closer contours (a lower ASSD with a mean of 1.61 ± 0.46 mm) than all other models. The DIRNet 4 achieved mean scores of 0.89 ± 0.02, 0.86 ± 0.04, and 1.61 ± 0.46 mm for the Dice, Jaccard, and ASSD, respectively, which are higher than other models (Tables 2, 3, and 4). To illustrate the model’s performance on each organ, the Marching Square algorithm [55] was used to generate contours from the 2D predicted binary masks for each DIRNet model (Fig. 7, blue contours).

**Table 4:** Average symmetric surface distance (ASSD) calculated over deformed pelvic masks and the ground-truth masks for the different variants of DIRNet models. Results are given for the entire DIRNet models for the cervix, bladder, and rectum

		Average symmetric surface distance (ASSD)		
		Cervix (mean ± SD)	Bladder (mean ± SD)	Rectum (mean ± SD)
Before registration		4.00 ± 1.82 mm	2.68 ± 1.09 mm	7.70 ± 3.90 mm
SimpleElastix		2.94 ± 0.78	3.26 ± 0.74	3.04 ± 1.50
DIRNet	Base model	1.85 ± 0.45	1.52 ± 0.86	1.97 ± 0.78
	2	2.63 ± 1.01	3.46 ± 0.90	3.70 ± 2.75
	3	2.86 ± 0.65	2.87 ± 2.37	2.92 ± 0.94
	4	**1.61 ± 0.46**	**1.17 ± 0.15**	**1.06 ± 0.42**

Among other models, the basic DIRNet model (DIRNet 1) achieved a higher score in terms of the Dice, Jaccard, and ASSD for the cervix. Furthermore, the DIRNet 3 exhibited a higher score, compared to the SimpleElastix based on ASSD and Jaccard scores. However, based on the Dice score, DIRNet 3 and SimpleElastix had the same performance on the cervix. More detailed results are summarized in Tables 2, 3, and 4 and illustrated in Fig. 7. Representative image slices from the input moving and fixed CT image pairs, together with the registered CT images, are provided in Fig. 7.

### Performance on Bladder

The DIRNet 4 network, trained with more Conv layers, showed a better performance with respect to the basic DIRNet model in terms of the Dice, ASSD, and Jaccard indices. This indicates that adding more Conv layers would significantly improve the performance of the model for all three organs. Furthermore, the basic DIRNet and DIRNet 4 models exhibited improved performance in image registration for the bladder. However, the remaining models failed to improve bladder deformation.

### Performance on Rectum

**Fig. 8:**
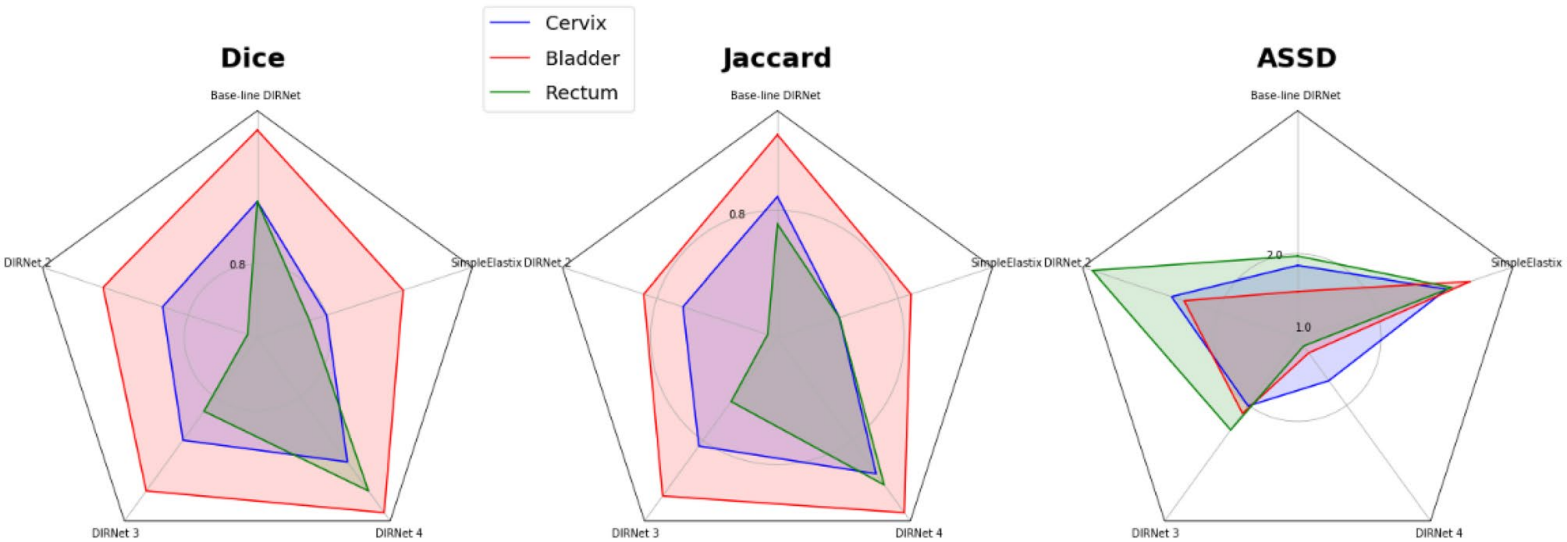
Radar plots of the quantitative metrics. Results are shown for three metrics, five DIRNet models, and three organs averaged over 15 patients. The colored polygon represents the organs (blue: cervix, red: bladder, and green: rectum). Each corner corresponds to a variant of DIRNet models. Each model has three scores (at the corners) for three organs. For the Dice measure (left plot), the DIRNet 4 has a greater dice score relative to all other DIRNet variants, except for the DIRNet 3 in the bladder. Similarly, for the Jaccard coefficient (middle plot), the DIRNet 4 has a greater score for all organs, in comparison with all other DIRNet variants. Regarding the average symmetric surface distance (ASSD) (right plot), the DIRNet 4 also has a lower score (best match) for all organs, and the DIRNet 3 has a similar ASSD score as the DIRNet 4 for the cervix

Based on the Dice, Jaccard, and average symmetric surface distance metrics, the DIRNet 4 model achieved a higher score for the rectum. Mean Dice, ASSD, and Jaccard coefficients of 0.93 ± 0.02, 1.06 ± 0.42, and 0.88 ± 0.04, respectively, were obtained from the DIRNet 4 model for the rectum. More detailed results are presented in Tables 2, 3, and 4. Additionally, a radar plot of the results was generated to show the models’ performance based on multiple quantitative metrics (Fig. 8). Representative slices from the input fixed and moving images, together with the registered image, are provided in Fig. 7 for qualitative visual assessment of the registration results based on the test data.

## Discussion

This study demonstrated the feasibility of deep learning-based DIR to account for inter-fraction organ motion in the HDR brachytherapy. This method exploits image similarity features between fixed and moving images to train a CNN model for the DIR. The CNN model was evaluated for pelvis CT images. Since there is no specific dataset (publicly available) for such an application, a deformation simulation was introduced considering realistic image deformation within the bladder and rectum organs. In the proposed method, DIRNet architecture, introduced by de Vos et al. [50], was considered the basic model. To evaluate the effect of the loss functions, Conv layer, and pooling layers, different types of the DIRNet model were trained and tested. All models were separately evaluated for all three organs (i.e., bladder, cervix, and bladder) using the binary masks obtained from the models’ output and the ground-truth data. For this purpose, the Dice, Jaccard, and average symmetric surface distance metrics were employed to assess the performance of deep learning-based image deformation within the specified organs. Furthermore, the synthetic deformation based on correlated stochastics field was applied to simulate an inter-fraction organ deformation.

To compare the proposed method to conventional intensity-based DIR algorithms, SimpleElastix was implemented on the dataset with tuned parameters. Results showed that the DIRNet 4 model that benefits from more Conv layers (eight layers, in comparison with the basic DIRNet model) achieved better results on all three organs; nevertheless, slightly increased errors were observed for the bladder due to the large deformation of this organ. It is suggested that the number of Conv layers has a significant effect on the performance of the model, in comparison with the average pooling layer. The reason is as Conv layers increase, the model can extract more discriminative features from the input images that help the transformation function to estimate better deformation parameters. On the other hand, the average pooling layer may decrease the image dimension which results in poor resolution in the deformation estimation field. However, for large image deformation within the rectum, pooling layers had a greater effect on the performance of the model, compared to the loss function. For medium deformation relative to the rectum, such as within the cervix, the loss function was important, in comparison with the pooling layer. The mean squared loss function, which magnifies the estimated errors, would show more sensitivity to small errors between the predicted and the ground-truth images. Therefore, the MSE loss function could not be a good choice for small deformations. Furthermore, for low deformation, such as bladder, most of the DIRNet models did not improve registration accuracy. However, DIRNet 1 model (the basic DIRNet model) improved bladder registration.

In comparison with similar studies on the rigid-registration problem, Miao et al. [37] used a CNN model to predict rigid transformation parameters for 2D/3D X-ray attenuation maps and 2D X-ray images of the volar plate for virtual implant planning systems. They proposed a hierarchical regression in which the six transformation parameters were categorized into three groups. By transforming the aligned data, synthetic ground-truth data was created. Their proposed methodology beat registration algorithms (by using mean target registration error (TRE)) based on gradient correlation (TRE: 0.315 mm), mutual information (TRE: 0.285 mm), and an optimization-based (TRE: 0.282 mm) method. In a study conducted by Eppenhof et al. [33], a 3D CNN was used to estimate deformable image transformation directly from two input images. To automatically annotate the ground-truth data for supervised training, they applied a random modification of aligned inhale-exhale 3D lung CT picture pairings. Synthetic deformations generated by a random transformation estimator were applied to images. They compared their approach against other state-of-the-art lung registration by measuring the registration error across 10 subjects with 300 landmarks. A mean TRE of 4.02 ± 3.08 mm across 10 subjects was achieved by this model. Fast registration time and automatic annotation are the most important aspects of their study. To enhance the diversity of the registration dataset, Sokooti et al. [41] used random DVFs to augment their dataset. They used a multi-scale CNN on intra-subject 3D chest CT images to estimate the DVF. They proposed a late fusion approach for the input data to the model. For single resolution, B-spline RegNet has a better TRE (4.39 ± 7.54 mm), in comparison with a single resolution with a TRE of 5.48 ± 7.56 mm. However, multi-resolution B-spline exhibited an improved TRE of 2.19 ± 6.22 mm.

Hu et al. [56] proposed an end-to-end CNN approach to predict the displacement field for multimodal image registration between multiple labeled structures. Furthermore, they addressed the challenges of the ground-truth generation for supervised learning by higher-level correspondence information for voxel-level labeling. They used 108 pairs of T2-weighted MRI and 3D transrectal US images for several network architectures. The mean TRE of 3.6 mm on landmark centroids and the median Dice of 0.87 on the prostate gland were achieved. In a study conducted by de Vos et al. [50], an end-to-end deep learning DIR called DIRNet was proposed. They trained a different variant of DIRNet on 69,540 cardiac cine MRI image pairs for the training and 63,840 image pairs for validation. They compared the results with the conventional intensity-based DIR (SimpleElastix) in terms of the Dice, MAD, and 95th SD metrics. In comparison to SimpleElastix, they concluded that a DIRNet model with overlapping patches and Conv layers before and after the pooling layer produced better results.

In the present experiment, it was found that the main advantage of this study was conducting deformation simulation based on anatomical changes by inserting applicators inside the vaginal cavity. This simulation also improved the proposed models through the use of binary masks which resulted in pixel-level accuracy for model evaluation. Future studies will aim to further improve the proposed method by adding more Conv layers and testing different loss functions. Although a dataset of 57 cervical patients was used in this study, increasing the training dataset would result in a model with improved accuracy and generalizability by using data augmentation [57] and decentralize federated learning [58] approaches. Another suggestion would be the use of more structure contours to take into account more anatomical considerations. Deformation simulation has a great effect on results; therefore, in future studies more sophisticated deformation models would be applied, and a variety of more patients may be investigated from different treatment strategies in terms of the type of applicators and the relevant anatomical deformation.

## Conclusion

In summary, a deep learning-based DIR was introduced in this study to take into account inter-fraction deformation in high-dose-rate cervical cancer BT. The trained network enables a fast and fully-automatic DIR algorithm using a pair of fixed and deformed images. The trained models were applied to 15 cervical cancer patients with manually defined labels for the bladder, cervix, and rectum. The deep learning-based registration results were compared to the SimpleElastix, which is a conventional intensity-based DIR algorithm. The proposed method outperformed the SimpleElastix in all three organs based on the Dice, Jaccard, and ASSD metrics. Finally, it could be concluded that DIRNet 4 model is able to consider large and low deformation for the rectum and bladder, respectively.

## Data Availability

Not applicable.

## Footnotes

1 https://matplotlib.org/

2 https://scikit-image.org/

3 https://numpy.org/

## Code Availability

Open-source library including TensorFlow used in this study.
